# Leakage Current Non-Uniformity and Random Telegraph Signals in CMOS Image Sensor Floating Diffusions Used for In-Pixel Charge Storage

**DOI:** 10.3390/s19245550

**Published:** 2019-12-16

**Authors:** Alexandre Le Roch, Vincent Goiffon, Olivier Marcelot, Philippe Paillet, Federico Pace, Jean-Marc Belloir, Pierre Magnan, Cédric Virmontois

**Affiliations:** 1 ISAE-SUPAERO, Université de Toulouse, F-31055 Toulouse, France; vincent.goiffon@isae-supaero.fr (V.G.); olivier.marcelot@isae-supaero.fr (O.M.); federico.pace@isae-supaero.fr (F.P.); pierre.magnan@isae-supaero.fr (P.M.); 2 CEA, DAM, DIF, F-91297 Arpajon, France; philippe.paillet@cea.fr; 3 Centre Nationale d’Etudes Spatiales (CNES), F-31055 Toulouse, France; jean-marc.belloir@cnes.fr (J.-M.B.); cedric.virmontois@cnes.fr (C.V.)

**Keywords:** CMOS image sensor, floating diffusion, sense node, leakage current, retention time, random telegraph signal, electric field enhancement

## Abstract

The leakage current non-uniformity, as well as the leakage current random and discrete fluctuations sources, are investigated in pinned photodiode CMOS image sensor floating diffusions. Different bias configurations are studied to evaluate the electric field impacts on the FD leakage current. This study points out that high magnitude electric field regions could explain the high floating diffusion leakage current non-uniformity and its fluctuation with time called random telegraph signal. Experimental results are completed with TCAD simulations allowing us to further understand the role of the electric field in the FD leakage current and to locate a high magnitude electric field region in the overlap region between the floating diffusion implantation and the transfer gate spacer.

## 1. Introduction

Trends in imaging manufacturing over the last decade have been a race for integration seeking a reduction of pixel pitch, an increase in pixel resolution, and an increase in pixel performances. Nowadays, as the successor of Charge-Coupled Devices (CCD), CMOS Image Sensors (CIS) comprise an array of active pixels. High-end consumer-grade CIS are based on the four-Transistor (4T) Pinned Photodiode (PPD) structures. 4T pixels include a photosensitive region (pinned photodiode), a charge transfer transistor, a sense node, a reset transistor, a source follower transistor for in-pixel amplification, and a column bus transistor. Generated charges are collected and converted to the voltage domain within each pixel, and ultimately readout. To enable both still and motion photography, in-pixel charge storage in Sense Node (SN) Floating Diffusions (FD) is considered in global shutter and burst CIS. Before being converted to the voltage domain, the charge retention time in the FD varies depending on the pixel position in the sensor array. Typically, the longest retention time can be as long as the array readout time. Consequently, the performances of CIS using in-pixel charge storage in FD are closely related to mechanisms occurring in the FD as it plays a significant role in the detection chain. As the FD leakage current is more influent when long storage times are concerned, the signal of the last pixels readout can be drastically impacted. The required long storage time in FD make CIS very sensitive to FD leakage current and FD leakage current Random Telegraph Signals (RTS) as reported in [1,2]. Still, the fundamental motivations for shrinking pixel size include increasing the resolution for a given camera module size, but it makes the in-pixel charge storage efficiency a prominent parameter to reach high pixel performances. Therein lies the fundamental challenge of CIS using long-duration charge storage in FD, such as CIS operated in global shutter mode or burst mode, as discussed in [3,4].

In this study, the leakage current non-uniformity, as well as the leakage current RTS, are investigated in the FD. This study provides another vision and complete other investigations such as [5] led on the output voltage temporal noise. Here, results point out that high magnitude electric field regions could explain the high FD leakage current non-uniformity and its fluctuation. Additionally, high magnitude electric field effects, such as transfer-gate-induced leakage current, are investigated to understand further the phenomena involved in the FD while giving new insights on the electric field enhancement of the charge generation mechanisms. Experimental results are combined with and compared to TCAD simulations allowing us to further understand the role of the electric field in the FD leakage current.

## 2. Experimental Details

**Figure 1:**
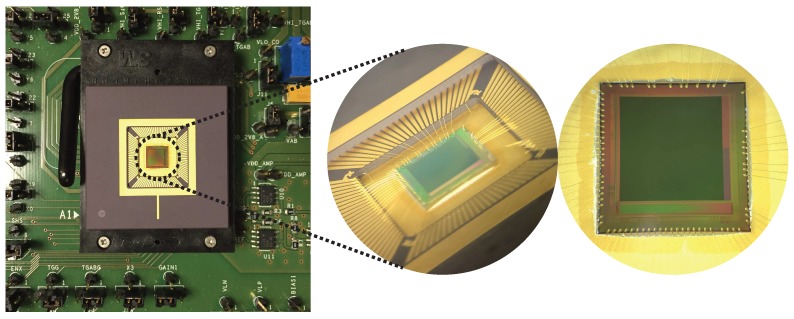
Pictures of the 4-Transistors (4T) Pinned Photodiode (PPD) CMOS Image Sensor (CIS) under test mounted on a PGA100 ceramic support.

**Figure 2:**
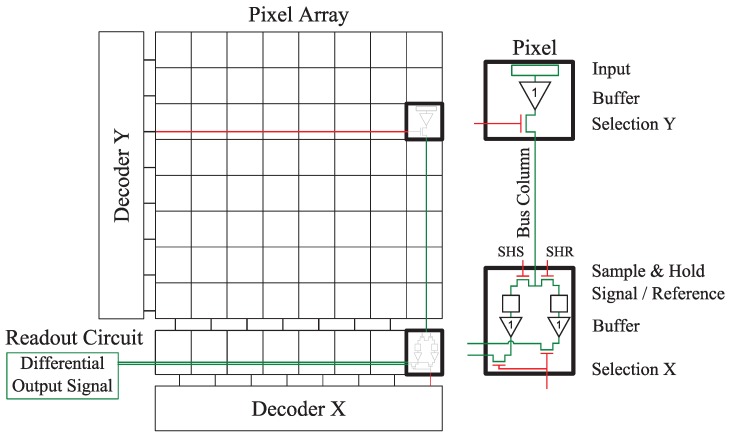
Illustration of the electrical architecture of the CMOS Image Sensor (CIS) under test. The red lines correspond to the decoder signals. The green lines correspond to the signal for a given pixel selected by the decoders.

**Figure 3:**
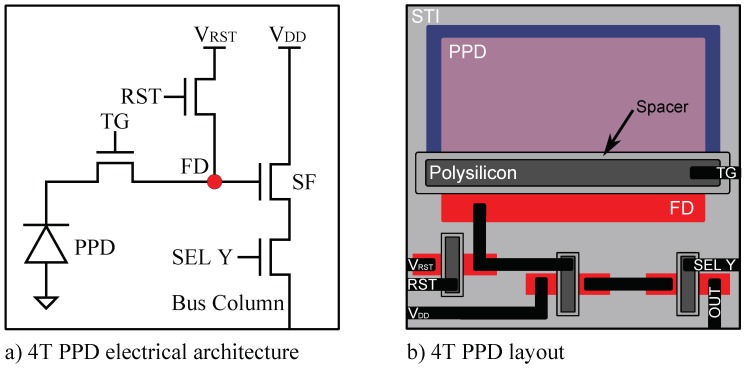
Illustration of the electrical architecture (**a**), and the layout (**b**) of the 4-Transistors (4T) Pinned Photodiode (PPD) pixel under test. In the layout illustration, the metal layers are in black, the PPD consists of a pinning layer in blue and a pinned layer in red. The cross-section presented in Figure 4 is based on the same colored layers.

**Figure 4:**
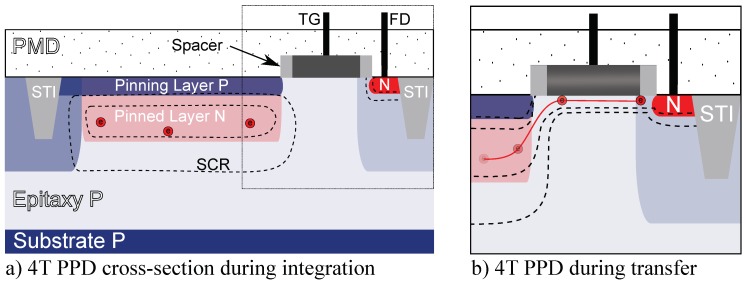
Illustration of the the 4-Transistors (4T) Pinned Photodiode (PPD) pixel cross-section in the integration configuration (**a**), and in the charge transfer configuration (**b**).

The CIS under test is a 512×512 4T PPD custom imager manufactured in a commercially available 180 nm CIS technology. The pixel array has a 7 μm pitch. The die is mounted on a PGA100 ceramic package and tested by using an electronic board as shown in Figure 1. The CIS architecture is illustrated in Figure 2. For each pixel, the FD signal is amplified within the pixel before being sampled and hold in the readout circuit. The output signal results from a second amplifier in the readout circuit. Pixels are organized in a 2D-array and can be accessed independently with the simultaneous activation of the corresponding line and column by the decoder circuits. At the pixel level, the electrical architecture, as well as the layout, are illustrated in Figure 3. In a 4T PPD CIS, the TG is kept at its low bias level during the integration. In this configuration, the PPD is isolated from the readout chain and collects the thermally generated charges constituting the PPD dark current, as well as the photo-generated charges in the case of illumination. An illustration of a 4T PPD pixel cross-section in the integration configuration is presented in Figure 4a. In this figure, the PPD Space Charge Region (SCR) is isolated from the FD. Before the end of the integration time, the FD is reset by applying a positive bias on the reset transistor gate (i.e., RST signal in Figure 3a). The FD is positively biased by the $V_{RST}$ bias via the reset transistor. After a few microseconds, the reset transistor gate is biased to 0 V, and the FD becomes a positively biased floating node. Following the FD reset, a FD sampling is performed, resulting in a FD signal amplification by the Source Follower (SF) transistor and a line selection by the selection transistor (SEL Y). The amplified FD signal is held in the readout circuit (SHR) as a reference sample. At the end of the integration time, the TG is positively biased and allows the collected charged to be transferred to the FD, reducing its potential. This configuration is called the charge transfer and is illustrated in Figure 4b. After charge transfer, a second FD sample and hold is performed in the readout circuit (SHS) as a signal sample. After a second amplification step and a column selection by the X decoder, the output signal is the difference between the signal sample and the reference sample. This technique is called Correlated Double Sampling (CDS) and allows canceling the reset kTC noise and reducing the 1/*f* noise in the FD. In the sensor under test, both the reference sample and the signal sample are stored in the readout circuit in a Metal Insulator Metal (MIM) capacitor structure.

**Figure 5:**
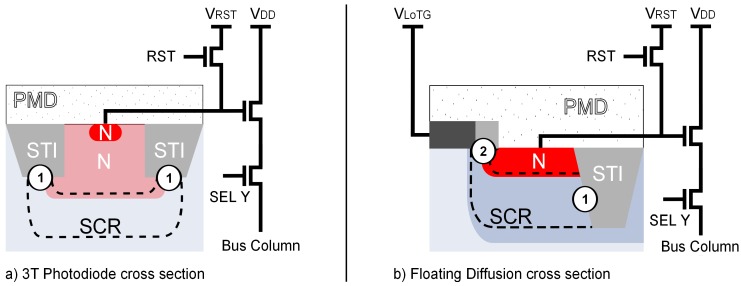
Cross-section illustrations depicting the structural differences between a conventional 3T photodiode (**a**) and a FD (**b**). $\mathrm{Si}/{\mathrm{SiO}}_{2}$ interface defects located in the depleted regions are referred to as ①. The Gate Induced Leakage (GIL) current is referred to as ②. SCR: Space Charge Region. PMD: Pre-Metal Dielectric. STI: Shallow Trench Isolation.

This study aims to investigate the charge storage in FD structure. To do so, the CIS is operated in the dark with a constant TG bias, kept at its low bias level. In this configuration, the PPD is isolated from the FD at all time, and the CIS remains in the configuration illustrated in Figure 4a. The CIS is operated as a standard 3T CIS where the sensed structure is a FD instead of a photodiode. By using a 3T chronogram to operate the CIS, the CDS is no longer possible since the node on which the reset is operated is the same as the one the charges are integrated. Double sampling is still performed with SHS and SHR, but it appears non-correlated because the two signals are not taken on the same frame. Therefore, the double sampling still reduces some readout noises, but it does not cancel the reset noise. Figure 5a shows the cross-section of a standard 3T CIS photodiode and Figure 5b the cross-section of a FD. A variable integration time is inserted between the signal and the reference samples corresponding to a FD integration time. This FD integration time is directly linked to the charge retention time in the case of a global shutter configuration as well as in the case of any application where the generated charges are stored in-pixel in a FD. Moreover, this CIS allows modifying both the TG bias (i.e., $V_{LoTG}$) as well the reset voltage (i.e., $V_{RST}$). Therefore, different bias configurations are studied to evaluate the impacts of such parameters on the charge storage retention time in FD. By using the pixel array, a multitude of FD structures are simultaneously studied, allowing considering a population of FD and studying the leakage current distribution evolution with $V_{LoTG}$ and $V_{RST}$ all over the sensor array. To explore the FD leakage current as well as the technological levers which can influence it, several bias conditions at room temperature have been tested by following both the FD leakage current non-uniformity as well as the FD leakage current RTS.

All the measurements have been performed in the dark in a temperature-controlled chamber at 22 ${}^{\circ }C$ ($\pm 1{}^{\circ }C$). The leakage current measurements use five different integration times from 136 ms (minimum readout time related to the size of the pixel array) to one second. Each image, attributed to a given integration time, is built from the average image of ten acquisitions to reduce the temporal noise. The dark current is computed as the slope of the output voltage as a function of the integration time using the Charge to Voltage factor of (CVF). The CVF has been computed with the mean–variance method [6] all over the sensor array and is estimated to 13.5 μV $/e^{-}$. The leakage current RTS analysis method uses a rising edge detection algorithm over 15,000 images with a one-second sampling time as introduced in [7] and further developed in [8]. From this detection method, the RTS maximum transition amplitude is measured as the highest transitions between two leakage current levels [9]. Therefore, each FD presenting a RTS leakage current behavior has a characteristic RTS maximum transition amplitude whose evolution is investigated with variable biasing conditions.

## 3. FD Leakage Current Non-Uniformity

**Figure 6:**
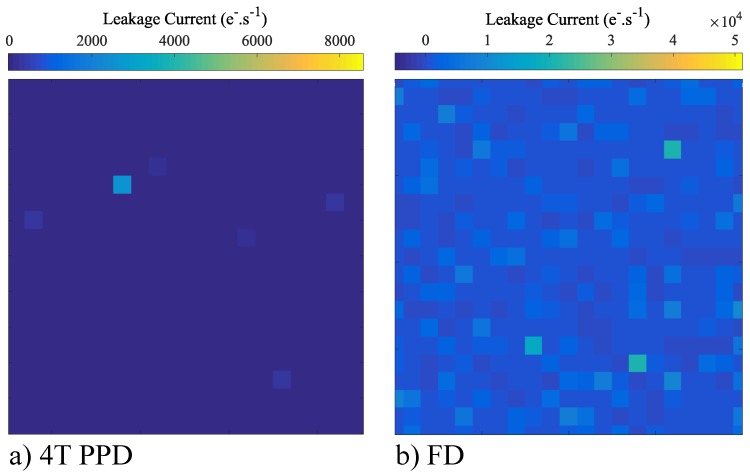
Leakage current over a small part of the sensor array in 4T-PPD (**a**) and in FD (**b**) ($V_{RST}\;=\;3.3\;V$; $V_{LoTG}=0\;V$).

The leakage current over a small and representative part of the sensor array both in the 4T-PPD and in the FD are reported in Figure 6a and Figure 6b respectively. In the FD, the leakage current is about three orders of magnitude higher than the one observed in the 4T-PPD. The observed FD leakage currents are even higher than those commonly observed in conventional 3T photodiode whose structure might seem similar [10]. To better understand the possible leakage current sources in FD, a comparison with the conventional 3T photodiode is proposed. As illustrated in Figure 5 depicting the cross-sections of a conventional 3T photodiode in Figure 5a and a FD in Figure 5b, the FD structure presents several particularities. Indeed, in the 3T photodiode structure, the doping profile of the photodiode n-implant is optimized (usually with p-well implantations) to locate the depleted region at the bottom of the STIs avoiding the STI sidewalls and its corners which is not the case in FD. When depleted, the STI sidewalls are known to be a high defect concentration region and have been identified as the dominant leakage current source in [11]. The results reported in Figure 6 suggest the existence of a multitude of generation centers located at the $\mathrm{Si}/{\mathrm{SiO}}_{2}$ interfaces like the TG oxide and the shallow trench isolation (STI) sidewalls which are directly in contact with the FD depleted regions. Moreover, whereas the doping level of CIS photodiodes is optimized to avoid unwanted high-magnitude electric field, the FD structure is based on a transistor source/drain implantation with a higher doping level at the junction. The higher doping level of the N-implantation into the p-well implantation in the FD compared to the 3T photodiode leads to a high-magnitude electric field within the FD. Indeed, the doping level at the junction in the FD is about $N_{D_{\mathrm{FD}}}\approx 10^{19}{\mathrm{cm}}^{-3}$ and reach $N_{D_{3T-\mathrm{PD}}}\approx 10^{17}{\mathrm{cm}}^{-3}$ in 3T photodiode.

**Figure 7:**
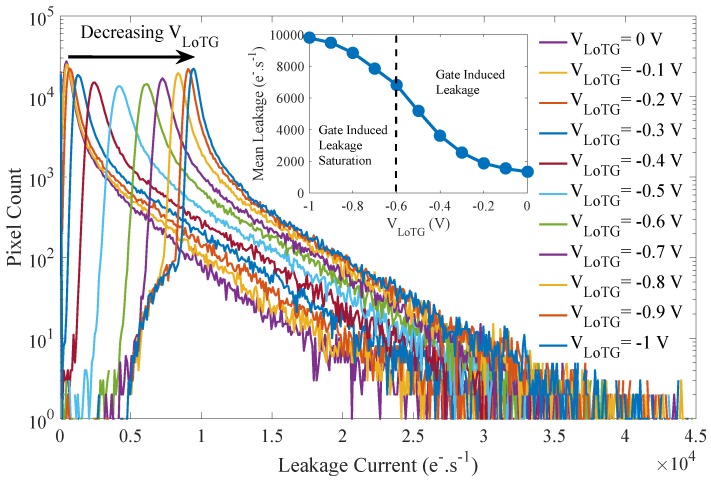
Evolution of the mean and the distribution of the FD leakage current with the $V_{LoTG}$ bias ($V_{RST}=3.3\;V$).

The mean and the distribution evolutions of the FD leakage current with the TG bias are visible in Figure 7. These results highlight a gate-induced leakage (GIL) current, which increases the mean leakage current with decreasing TG bias as already observed in [12,13]. This GIL current is related to the TG overlap with the N-implant referred to as ② in Figure 5. Regarding the leakage current distribution, this GIL current leads to a shift of the entire histogram toward higher leakage current without any change in the leakage current distribution shape. The impact of the TG-induced electric field on the leakage current results in a uniform leakage current increase all over the sensor array. It empathizes that the TG low-level bias has to be considered for long duration charge storage in FD.

**Figure 8:**
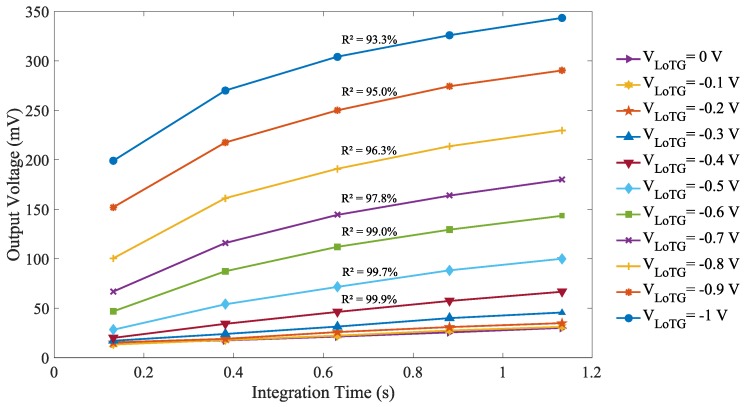
Evolution of the output voltage of a single representative FD of the sensor array with the integration time for different $V_{LoTG}$ bias ($V_{RST}=3.3\;V$).

The evolution of the output voltage of a single representative FD with the integration time for different TG bias is reported in Figure 8. With decreasing TG bias, the leakage current (i.e., proportional to the slope) is increasing as already observed in Figure 7. The FD leakage linearity is followed by the linear correlation factor referred to as $R^{2}$ in Figure 8. The induced TG electric field degrades the FD leakage linearity for TG bias lower than -0.4 V and increases as the TG bias decreases. This non-linearity could be explained by the reduction of the field-assisted GIL current with decreasing FD potential during the charge integration. For low GIL current corresponding to TG bias higher than -0.4 V, no significant FD leakage non-linearity is observed. The observed non-linearity is only observed for the FD leakage current and does not apply on photo-generated charges. Therefore, in the case light integration, a good FD linearity can be expected. Additional results not presented here indicate that the reset voltage variation does not induce any non-linearity in the FD leakage current.

**Figure 9:**
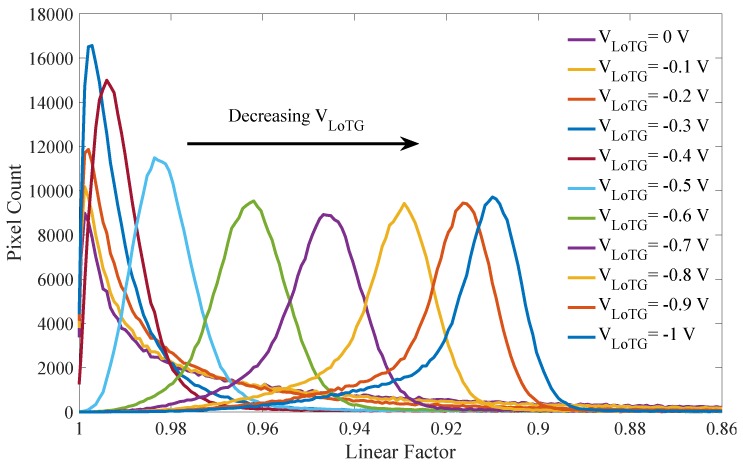
Evolution of the FD linear factor histogram over the sensor array with the $V_{LoTG}$ bias ($V_{RST}=3.3\;V$).

**Figure 10:**
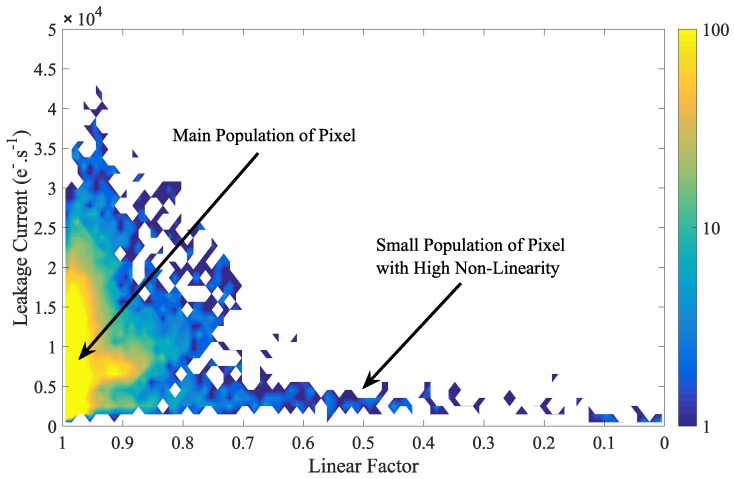
Two dimensions histogram showing the leakage current as a function of the FD linear factor ($V_{RST}=3.3\;V$; $V_{LoTG}=0\;V$).

The evolution of the FD leakage non-linearity all over the sensor array with the TG bias is reported in Figure 9. As seen on the distributions, the majority of the FD over the sensor array are impacted by the TG-induced leakage non-linearity. It empathizes that the TG low-level bias has to be considered for long-duration charge storage in FD. Figure 10 shows the two dimensions histogram showing the FD leakage current as a function of the linear factor at $V_{LoTG}=0\;V$. The main population of pixel discloses a good leakage linearity as observed in Figure 9 at $V_{LoTG}=0\;V$.

**Figure 11:**
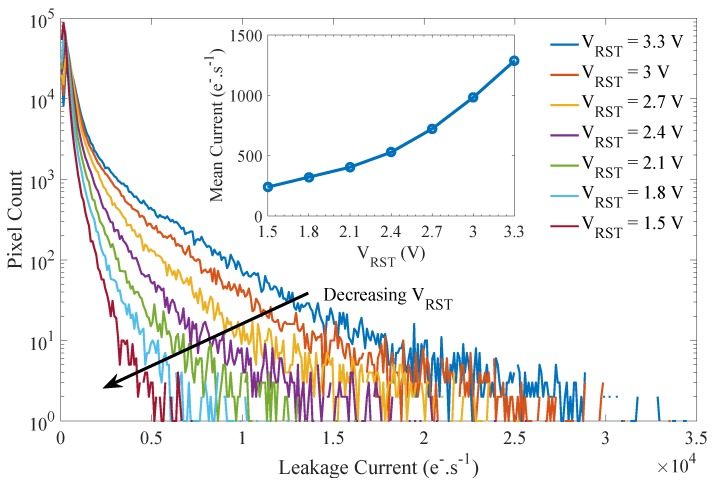
Evolution of the mean and the distribution of the FD leakage current with the $V_{RST}$ bias ($V_{LoTG}=0\;V$).

The evolution of the mean and the distribution of the FD leakage current with the reset voltage is reported in Figure 11. The mean leakage current decreases with the decreasing reset voltage for which the linearity of the electrical transfer function is ensured (i.e., until $V_{RST}=1.5\;V$). Regarding the FD leakage current distribution, the electric field induced by the reset voltage only impacts the leakage current tail conserving the same leakage current for the majority of the FD over the sensor array. The leakage current tail is extended with increasing reset voltage. These results suggest the existence of an electric field enhancement (EFE) of generation centers located in high magnitude electric field regions corresponding to a small population of FD as discussed in [14,15]. This evolution, which cannot be highlighted by the mean leakage current evolution, cannot be attributed to a simple depleted region extension since it would have led to a shift of the whole histogram. Therefore, the FD electric field distribution into the FD volume also needs to be addressed to ensure long duration charge storage in FD.

## 4. FD Leakage Current RTS

**Figure 12:**
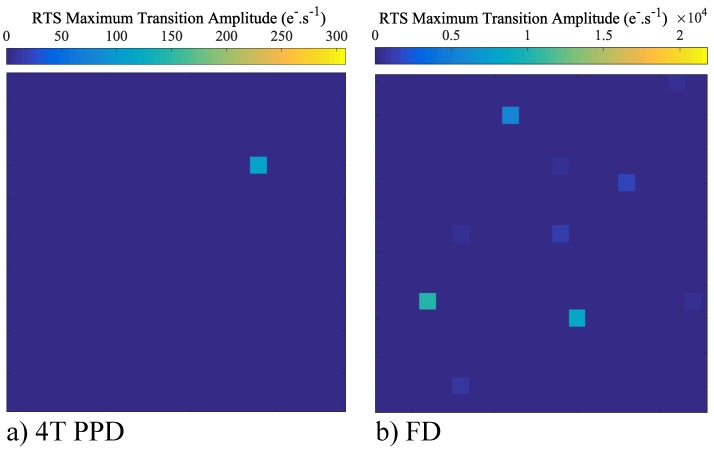
RTS maximum transition amplitude over a small part of the sensor array in 4T-PPD (**a**) and in FD (**b**) ($V_{RST}\;=\;3.3\;V$; $V_{LoTG}=0\;V$).

**Figure 13:**
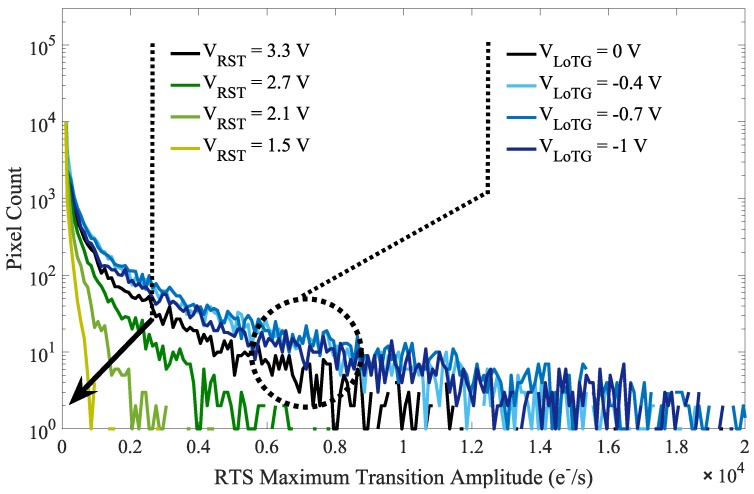
Histograms of the RTS maximum transition amplitude in FD over the sensor array for different bias conditions. The impact of $V_{LoTG}$ is followed with $V_{RST}=3.3\;V$ and the impact of $V_{RST}$ is followed with $V_{LoTG}=0\;V$.

**Figure 14:**
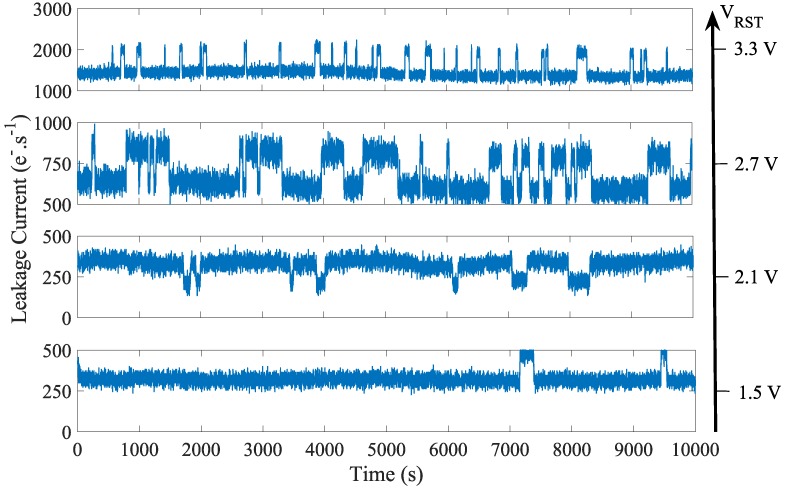
Leakage current evolution with time of on single pixel for different reset voltages operated with $V_{LoTG}=0\;V$.

The leakage current RTS over a small and representative part of the sensor array both in the 4T-PPD and in the FD are reported in Figure 12a and Figure 12b respectively. Compared to the PPD, the FD reveals a higher number of RTS pixels. Moreover, in the FD, the RTS maximum transition amplitudes are higher than those observed in the PPD. The histograms of the RTS maximum transition amplitudes in FD over the sensor array for different bias conditions are reported in Figure 13. The TG-induced electric field has a weak impact on the RTS maximum transition amplitude distribution. However, decreasing $V_{RST}$ clearly reduces the RTS distribution tail. These results suggest that the defects responsible for the RTS behavior in FD are impacted by the reset voltage and are more likely located at the STI sidewalls. The observed RTS amplitude enhancement is also reported in Figure 14 showing the leakage current evolution of a FD presenting an RTS center impacted by the reset voltage. In Figure 14, the depletion extension explains the mean leakage current increase with the reset voltage. Contrary to the leakage current value, accounting for each leakage current sources in a given FD deleted region, the maximum transition amplitude extracts the most significant leakage current transition for each FD and represents the contribution of one single RTS center. Therefore, the RTS maximum transition amplitude increase with the reset voltage cannot be attributed to a simple depletion region extension in the FD, but to an electric field enhancement of the RTS center amplitude. However, other mechanisms might occur in some FD. As the reset voltage increases, the extension of the depleted region in the FD might reach new RTS centers not present in the depleted region at lower reset voltage. This new RTS center can either turn a FD into a RTS-FD, or lead to a different RTS behavior if its amplitude is the highest transition in the given FD depleted volume. As previously introduced in Figure 11 for the leakage current, the results highlight the role of the reset-voltage-induced electric field into the FD PN junction both on the leakage current and the RTS amplitudes. Therefore, the reset voltage also need to be considered when considering long-duration retention time in FD.

**Figure 15:**
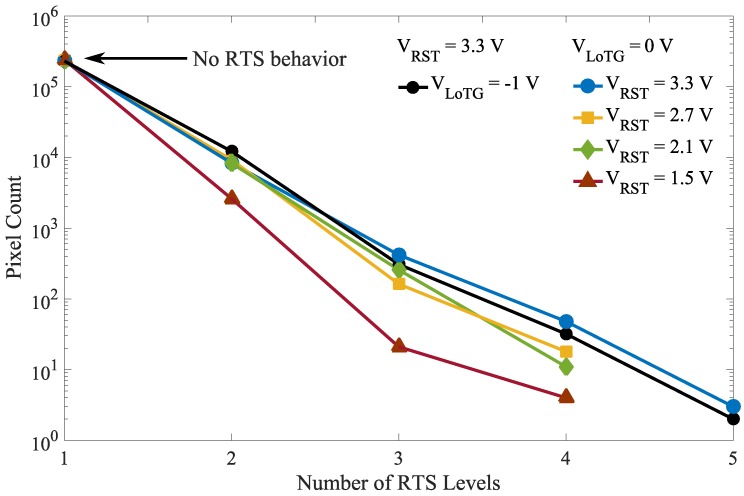
Histograms of the number of RTS level in FD for different TG bias with $V_{RST}=3.3\;V$ and reset voltages with $V_{LoTG}=0\;V$.

Figure 15 shows the RTS levels histogram evolution for different bias conditions. These results show that the electric field induced by the reset voltage increases the number of detected RTS levels, probably due to the RTS amplitude enhancement or the activation of RTS centers. As mentioned for the leakage current, it suggests the existence of high magnitude electric field regions, which play a significant role in the FD leakage current RTS. Moreover, it appears that the TG bias evolution does not induce any RTS level change in the FD, which confirms the assumption made from the RTS maximum transition amplitude distribution evolution in Figure 13 stating that the defects responsible for the RTS behavior in FD are more likely located at the STI sidewalls.

## 5. TCAD Simulation

**Figure 16:**
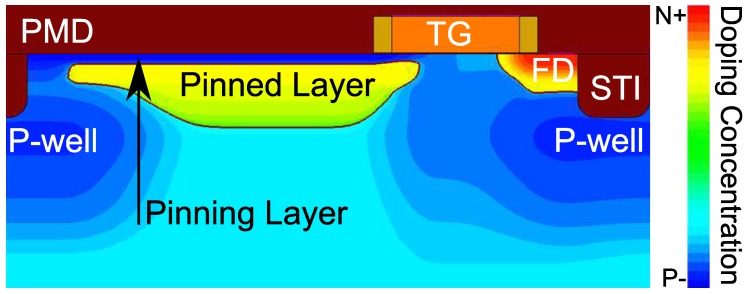
TCAD simulation of the doping distribution of the simulated device. In the color scale, blue parts are p-doped regions and red parts are n-doped regions.

2-dimensional TCAD simulations for different bias conditions are performed on a similar 4T PPD pixel structure to the one experimentally studied and visible in Figure 16 showing the doping distribution concentration. The doping distribution is computed from Secondary Ion Mass Spectrometry (SIMS) profiles employing the Sentaurus Structure Editor tool. Since the process flow is unknown, the simulated structure does not reproduce the real TG vicinity accurately, especially, the presence of additional implantations and, eventually, the spacer thickness and depth. Hence, the computed electric field in the FD overlap region may differ from the one present during the experimental work. However, the TCAD simulations can be used for relative comparison between the various bias conditions and for the location of the high magnitude electric field regions. The electrical simulations are performed using the Sentaurus Device software. Three electrical contacts are used: TG, FD, and the ground. In order to simulate the FD as a floating node, a reset transistor simulated in a mixed-mode is added to the FD contact as illustrated in Figure 3 where the drain of the reset transistor is biased at $V_{RST}$. For each simulation corresponding to a constant TG and reset bias, the reset gate bias varies from 0 to 3.3 V to bias the FD. Then, the reset gate bias return to 0 V, allowing the FD to be assimilated to a floating node. The reset voltage $V_{RST}$ varies from 1.5 to 3.3 V, and the TG bias $V_{LoTG}$ varies from 0 to -1 V.

**Figure 17:**
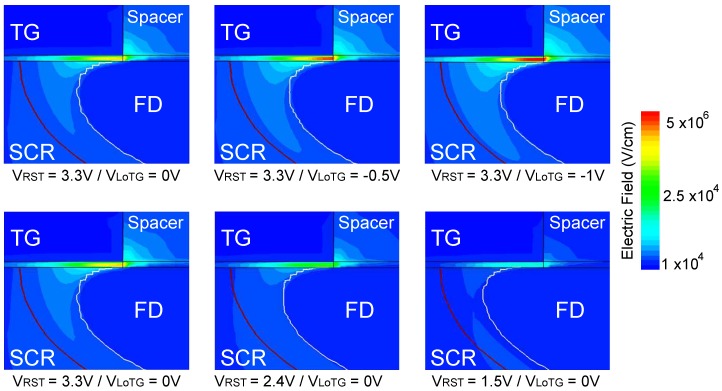
Electric field distribution evolution into the FD structure for the different bias conditions.

**Figure 18:**
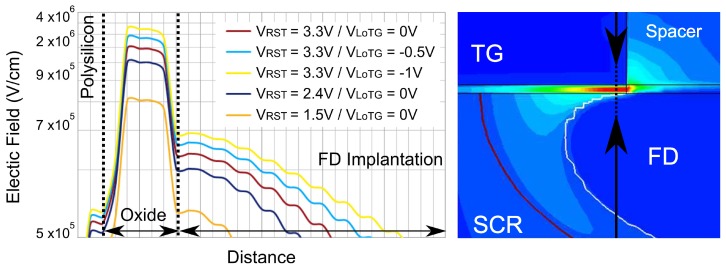
Electric field profile evolution for the different bias conditions in the overlap region between the FD implantation and the TG.

Figure 17 shows the electric field distribution into the FD structure. The upper part of the figure reports the electric field changes with decreasing $V_{LoTG}$ from 0 to -1 V at $V_{RST}=3.3V$. As the TG bias decreases, a high magnitude electric field located in the overlap region appears. The lower part of the figure reports the electric field changes with decreasing $V_{RST}$ from 3.3 to 1.5 V at $V_{LoTG}=0\;V$. As the reset voltage decreases, the electric filed in the overlap region decreases. These results highlight a high magnitude electric field region in the overlap area linked to the potential difference between the FD and the TG. This high magnitude electric field region explains the GIL current observed experimentally in the FD as the TG bias decreases. Figure 18 reports the electric field profiles in the overlap region for different bias conditions. The maximum electric field is located in the TG oxide and the electric field at the interface can reach $6\times 10^{5}$ V cm^−1^ to $7\times 10^{5}$ V cm^−1^ in the worst case, making the GIL current the principal responsible for the FD leakage current.

**Figure 19:**
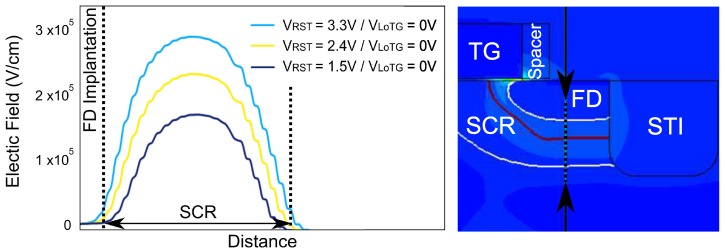
Electric field profile evolution for the different bias conditions into the FD implantation.

Figure 19 shows the electric field profiles along the FD depleted region with decreasing $V_{RST}$ at $V_{LoTG}=0\;V$. In the nominal operating conditions (i.e., $V_{RST}=3.3V$), the maximum electric field reach $3\times 10^{5}$ V cm^−1^ and achieve $1.5\times 10^{5}$ V cm^−1^ at $V_{RST}=1.5V$. Even if the reset voltage variations impact the maximum electric field into the FD implantation, the extension of the depleted region is not significant due to the p-well implantation bellow the STI visible in Figure 16. These results confirm that RTS centers present into the FD depleted region at the STI sidewalls can be impacted by an electric field whose magnitude can be up to twice more important between a reset voltage at $V_{RST}=1.5V$ and $V_{RST}=3.3V$. Moreover, the weak change in the depleted region extension of the FD depleted region confirms that the increase of the RTS maximum transition amplitude is mostly due to an EFE.

## 6. Conclusions

A main conclusion can be withdrawn for FD leakage current in CMOS Image Sensors: the GIL current is the main contributor to the overall leakage current, whereas the main mechanism that enhances the RTS amplitude is the electric field induced by the reset voltage. The experimental results, as well as the TCAD simulations, empathize the existence of high magnitude electric field regions in the FD overlap region, inferring that the FD electric field distribution must be addressed for CMOS Image Sensors using long-duration charge storage in FD such as CMOS Image Sensors operated in global shutter mode or burst mode. The solution to limit RTS in the FD could be the use of an enclosed layout transistor to isolate the FD depleted region from the STI sidewalls and the most virulent RTS centers. Since such enclose layout transistor would lead to a significant increase of the overlap area, applying a weak transfer gate bias would be essential to limit the GIL current.
